# Plasma miR-19b, miR-34a, and miR-146a expression in patients with type 2 diabetes mellitus and cataract: A pilot study

**DOI:** 10.17305/bb.2023.9933

**Published:** 2024-06-01

**Authors:** Adina Iuliana Milcu, Flavia Medana Anghel, Mirabela Romanescu, Aimee Rodica Chis, Andrei Anghel, Ovidiu Boruga

**Affiliations:** 1 Doctoral School, “Victor Babes” University of Medicine and Pharmacy, Timisoara, Romania; 2 Discipline of Ophthalmology, Department of Surgery I, “Victor Babes” University of Medicine and Pharmacy, Timisoara, Romania; 3 Department of Ophthalmology, Municipal Emergency Clinical Hospital, Timisoara, Romania; 4 Discipline of Biochemistry, Department of Biochemistry and Pharmacology, “Victor Babes” University of Medicine and Pharmacy, Timisoara, Romania; 5 Center for Complex Network Science, “Victor Babes” University of Medicine and Pharmacy, Timisoara, Romania

**Keywords:** Cataract, diabetes mellitus (DM), diabetic retinopathy (DR), microRNAs (miRNAs), biomarker, qRT-PCR

## Abstract

Cataract is among the most common ocular complications in diabetes mellitus (DM). While microRNA (miRNA) dysregulations in DM have been previously reported, consensus is still lacking concerning miRNA expression in cataract. Furthermore, the miRNA profile in diabetic cataract patients remains largely unexplored, and data on plasma expression levels are limited. Our study aimed to assess the plasma levels of three distinct miRNA species (hsa-miR-19b, hsa-miR-34a, and hsa-miR-146a) implicated in the development of cataract and/or DM. We investigated the circulating miRNA expression in DM patients diagnosed with cataract, compared to a non-DM cataract group. We employed qRT-PCR for relative quantification experiments and subsequently conducted a correlation analysis between miRNA expression levels and clinical characteristics. Our findings reveal that hsa-miR-34a and hsa-miR-146a are differentially expressed in the two cohorts. However, no significant correlation was observed between the clinical variables and miRNA levels. In summary, our results suggest a potential role for hsa-miR-34a and hsa-miR-146a in the biology of diabetic cataract.

## Introduction

Cataract is the worldwide second largest cause of treatable visual impairment [1] and the leading cause of blindness [2]. It is defined as a reduction in the transparency of the crystalline lens as a consequence of the accumulation of aggregated misfolded proteins [3, 4]. Cataractogenesis is a multifactorial process [5], associated with several systemic diseases, including diabetes mellitus (DM) [6]. The diagnosis is based on the visual acuity test and the retinal images obtained from biomicroscopy (slit-lamp exam) or ophthalmoscopy (funduscopic exam) [7, 8]. The only effective treatment is surgical removal of the opaque lens and implantation of an intraocular lens [9], a quick and safe procedure which dramatically improves patients’ vision and quality of life [10, 11].

Among all systemic diseases that have an impact on cataractogenesis, DM remains one of the most significant risk factors for lens opacification [12]. Conversely, cataract is one of the most common ocular complications in DM [13]. Different types of mechanisms have been proposed for DM-related cataractogenesis, such as dysregulations in the polyol pathway [14], osmotic and oxidative stress [6], and non-enzymatic glycation [15]. Earlier age onset of DM is associated with an increased risk of developing diabetic retinopathy (DR) [16], and diabetic patients who suffer from cataract are at a higher risk of developing DR [17]. Furthermore, cataract formation can hinder the visualization of the macula and retina, thus masking the level of DR and resulting in treatment difficulties [1].

MicroRNAs (miRNAs) are short non-coding RNAs that post-transcriptionally modulate gene expression at the mRNA level [18] and regulate various cellular processes [19]. In most cases, miRNAs suppress gene expression by interacting with the 3′ UTR of target mRNAs or, occasionally, with other regions, such as 5′ UTR, coding sequence, and gene promoters [20]. One miRNA can alter the stability of up to thousands of different mRNAs, while an individual mRNA can be targeted by multiple distinct miRNAs [21]. Nowadays, it is acknowledged that miRNA dysregulation is a causative factor in the progression of a wide range of human diseases [22]. Tissue miRNAs are routinely secreted in body fluids (plasma, serum, urine, and bile), where they show remarkable stability [20, 23], giving them a high potential to be used as diagnostic and prognostic biomarkers [24].

The implication of miRNAs in DM pathogenesis has already been documented [25–27], and studies have also identified several dysregulated miRNAs in cataract [28, 29]. Most of the research groups have investigated miRNA expression in lens cells or in aqueous humor (AH) – an invasive but useful approach for understanding how miRNAs mediate the development of cataract [30–33]. There is a surprising scarcity of data regarding the association of cataract with circulating miRNAs, although serum/plasma collection is less invasive [34]. Furthermore, the miRNA profile in diabetic cataract patients has only been vaguely investigated [35, 36]. Thus, further research is needed in order to fully comprehend the relationship between DM, cataract, and miRNAs. Besides, the identification of specific and non-invasive biomarkers (such as plasma miRNAs) would be a valuable asset in achieving early diagnosis and better management of the disease.

Hsa-miR-19b, hsa-miR-34a, and hsa-miR-146a are miRNA species that have been previously linked to DM and/or cataract. The three miRNAs are differentially expressed in various ocular tissues: miR-19b is dysregulated in retinal epithelial cells and AH [37, 38], miR-34a level correlates with the severity of lens opacification and age [39, 40], and miR-146a appears deregulated in diabetic lens, cornea, retina, and AH [35, 41–43]. However, data on their plasma expression and potential inference with the occurrence of DM in cataract are missing.

In this study, we aimed to determine to what extent the levels of has-miR-19b, hsa-miR-34a, and hsa-miR-146a are influenced by the presence of DM and whether this impacts the features of cataract. We used the qRT-PCR technique to investigate the expression profile of the three circulating miRNAs in a group of cataract patients with associated DM (diabetes mellitus cataract group [DMCG]), compared to a non-DM cataract group (cataract group [CG]).

## Materials and methods

### Study design and settings

**Figure 1.**
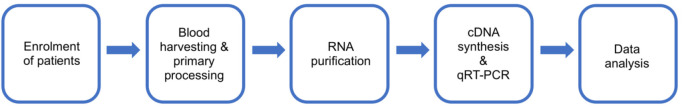
**Experimental rationale and procedures.** cDNA: Circular DNA.

The study explores the profile of three circulating miRNA species in patients with DM and cataract (case group – DMCG), compared with non-diabetic cataract patients (control group – CG). Blood specimens were collected and the plasma miRNA levels were quantified using the qRT-PCR technique. Experimental rationale and procedures are systematically presented in Figure 1.

### Participants/patient enrollment

All subjects have been enrolled through the Ophthalmology department/unit of the Timisoara Municipal Emergency Clinical Hospital and have provided their informed consent. Inclusion criteria were the following: documented diagnosis of cataract that required lens surgery (both groups), documented medical history of type 2 DM (case group), and provision of informed consent (both groups). Exclusion criteria included cognitive impairment and the inability to sign the informed consent.

The study enrolled a total number of 79 eligible patients suffering from advanced cataract that required lens surgery. The diagnosis of cataract was established by a certified ophthalmologist based on the visual acuity test and the biomicroscopic examination of the anterior segment of the eyeball. Clinical and biomicroscopic exams pinpointed specific diagnostic criteria: impaired visual acuity (with and without glasses), slow reduction in the clarity of vision, and opacification of the crystalline lens. The case group included patients with a documented history of type 2 DM, established by a specialist prior to admission to the Ophthalmology clinic. The diagnostic criteria included: fasting glycemia higher than 125 mg/dL, and glycated hemoglobin – HbA1c higher than 6.5%. The demographics and clinical characteristics of the individuals enrolled in the study are presented in Table 1.

**Table 1:** Demographics of the two cohorts

	**Case group (DMCG)**	**Control group (CG)**
Enrolled patients, *n*	50	29
Eligible plasma specimens, *n*	27	23
Gender ratio, males/females	11/16	7/16
Age (years), mean	69.5 ± 6.2	71.0 ± 7.6
Visual acuity (decimal value), mean	0.31 ± 0.19	0.34 ± 0.18
Intraocular pressure (mmHg), mean	13.6 ± 2.0	14.3 ± 2.7
Fasting glycemia (mg/dL), mean	133.1 ± 20.7	–

Data are presented as mean ± standard deviation. DMCG: Diabetes mellitus cataract group; CG: Cataract group.

### Blood harvesting and primary processing

Blood specimens were harvested one day prior to the surgical removal of the lens. The peripheral blood samples were obtained through puncture of the cubital vein, collected in EDTA-coated vacutainers, and refrigerated at 4 ^∘^C. Within the next 2 h, blood samples were centrifuged for 10 min at 1250 rpm, and the plasma fraction was aliquoted and stored at −80 ^∘^C, until further use. All plasma specimens with signs of hemolysis, turbidity, hyperlipidemia, or hyperbilirubinemia were discarded, to avoid interferences with downstream analyses.

**Figure 2.**
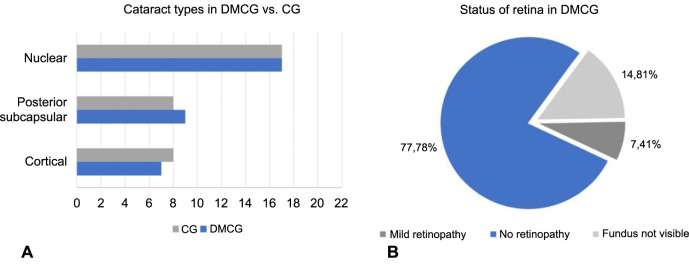
**Patient’s characteristics among the two groups**. (A) Cataract types in case (blue) vs control (gray) group; (B) Status of retina in case group. CG: Cataract group; DMCG: Diabetes mellitus cataract group.

### RNA purification

After thawing, plasma specimens were centrifuged for 5 min at 3000 rpm and 4 ^∘^C to eliminate precipitates and cell debris contaminants. Total RNA was extracted from a fixed plasma volume (200 µL), using the miRNeasy Serum/Plasma kit (Qiagen, Hilden, Germany), according to the manufacturer’s protocol. *Caenorhabditis elegans (C. elegans)* miR-39 miRNA mimic was used as a spike-in control for external normalization. The purified RNA samples were stored at −80 ^∘^C for downstream applications.

### cDNA synthesis and qRT-PCR

All reverse transcription and real-time PCR amplification reactions were performed individually for hsa-miR-19b, has-miR-34a, has-miR-146a, and cel-miR-39. We used the TaqManTM MicroRNA Reverse Transcription Kit (Thermo Fisher, Waltham, MA, USA) and specific stem-loop primers, provided by pre-designed inventoried TaqMan^TM^ MicroRNA Assays (Thermo Fisher, Waltham, MA, USA) for cDNA synthesis. The reaction mixture and the thermal cycler were set up according to the manufacturer’s instructions. After cDNA synthesis, the samples were stored at −20 ^∘^C until further use. For the amplification step, we performed individual qRT-PCR reactions and used dedicated inventoried TaqMan^TM^ MicroRNA Assays and TaqMan^TM^ Universal Master Mix II, no UNG (Thermo Fisher, Waltham, MA, USA), according to the manufacturer’s protocol. All qRT-PCR reactions were performed in triplicate.

### Variables and measurements

The measured quantitative variables were age, visual acuity, and intraocular pressure. Fasting glycemia was also determined for DMCG. Arithmetic mean and standard deviation were calculated and recorded separately for each group. Three categorical variables were used for stratification: gender, status of retina, and cataract subtype (depending on the localization of opacification).

To identify possible differences within groups, the cohorts were divided into subgroups, based on the retinal status and cataract subtype. Both the entire cohort and the subgroups were analyzed subsequently. For plasma miRNAs, fold changes were calculated using the 2^−ΔΔCT^ method of relative quantification with exogenous normalization to *C. elegans* miR-39 miRNA spike-in. Clinical variables (age, visual acuity, intraocular pressure, and fasting glycemia) and miRNA levels were subjected to correlation analysis.

To address potential sources of bias, clinical data were recorded anonymously. Statistical analysis was performed blinded, with encrypted cohort type (CG or DMCG), participants’ gender, and analyzed miRNA.

### Ethical statement

The local Institutional Ethical Review Board has evaluated and approved the present case-control study, which was carried out in accordance with the Declaration of Helsinki Code of Ethics (Nr. 35/04.04.2022).

### Statistical analysis

We checked the normalized Ct values for normality of distribution beforehand, using a Kolmogorov–Smirnov test. The statistical significance of the differentially expressed miRNAs in the two groups was determined using a two-tailed, unpaired, heteroscedastic student’s *t*-test. Correlation analyses were performed using a two-tailed Spearman test. For all tests, the statistical cut-off was 0.05. All statistical calculations were performed using Prism 8 for MacOS.

**Table 2:** Correlation matrix between the microRNA expression profile and the clinical characteristics in the case group (diabetes mellitus cataract group)

**Variables**	**Age**	**Visual acuity**	**Intraocular pressure**	**Fasting glycemia**	**miR-19b normalized Ct values**	**miR-34a normalized Ct values**	**miR-146a normalized Ct values**
Age		0.1178 (0.5586)	−0.2902 (0.1420)	0.4202 (0.0291)	0.1291 (0.5211)	−0.1643 (0.4127)	−0.0552 (0.7845)
Visual acuity	0.1178 (0.5586)		−0.2630 (0.1851)	−0.3091 (0.1167)	−0.0976 (0.6281)	−0.0805 (0.6900)	0.0257 (0.8987)
Intraocular pressure	−0.2902 (0.1420)	−0.2630 (0.1851)		0.1073 (0.5943)	0.1219 (0.5448)	0.2480 (0.2122)	0.1767 (0.3780)
Fasting glycemia	0.4202 (0.0291)	−0.3091 (0.1167)	0.1073 (0.5943)		0.0721 (0.7209)	0.1371 (0.4952)	0.0922 (0.6472)
miR-19b normalized Ct values	0.1291 (0.5211)	−0.0976 (0.6281)	0.1219 (0.5448)	0.0721 (0.7209)		0.3846 (0.0476)	0.5171 (0.0057)
miR-34a normalized Ct values	−0.1643 (0.4127)	−0.0805 (0.6900)	0.2480 (0.2122)	0.1371 (0.4952)	0.3846 (0.0476)		0.7784 (0.0000)
miR-146a normalized Ct values	−0.0552 (0.7845)	0.0257 (0.8987)	0.1767 (0.3780)	0.0922 (0.6472)	0.5171 (0.0057)	0.7784 (0.0000)	

Values represent Spearman correlation coefficient (*P* value). Ct: Quantification threshold.

**Table 3:** Circulating microRNA expression profile in case (DMCG) vs control (CG) groups before and after stratification

	**hsa-miR-19b**			**hsa-miR-34a**			**hsa-miR-146a**		
	**2^−ΔΔCT^**	**FC**	***P* value**	**2^−ΔΔCT^**	**FC**	***P* value**	**2^−ΔΔCT^**	**FC**	***P* value**
DMCG vs CG (All)	0.4510	0.7315	0.0740	−0.8700	1.8277	0.0158	−1.0229	2.0320	0.0278
DMCG vs CG (Cortical)	−0.0265	1.0185	0.9656	−0.5010	1.4152	0.4810	−0.7292	1.6577	0.4413
DMCG vs CG (Posterior subcapsular)	0.9105	0.5320	0.0515	−1.0210	2.0293	0.1602	−1.2063	2.3075	0.1582
DMCG vs CG (Nuclear)	0.3506	0.7842	0.2225	−0.7702	1.7055	0.0666	−0.8643	1.8204	0.1264
DMCG vs CG (No retinopathy)	0.4937	0.7102	0.0744	−0.9366	1.9140	0.0149	−1.0526	2.0742	0.0350
DMCG vs CG (Mild retinopathy)	0.1032	0.9309	0.9369	−1.8341	3.5655	0.3822	−1.1431	2.2086	0.7033
DMCG vs CG (Fundus not visible)	0.3702	0.7737	0.5404	0.2611	0.8345	0.7463	−0.7253	1.6532	0.4951

CG: Cataract group; DMCG: Diabetes mellitus cataract group; FC: Fold change.

## Results

### Patients’ characteristics

We found no statistically significant differences between the two cohorts regarding age (*P* ═ 0.4578), visual acuity (*P* ═ 0.5808), and intraocular pressure (*P* ═ 0.3031), indicating a homogenous sub-population. We categorized the cataract subtypes based on the localization of opacification (Figure 2A). Notably, a patient can suffer from more than one subtype. Nuclear cataract is the most prevalent cataract subtype among our patients, in both groups. Next, we checked for intracohort correlations between the clinical characteristics: age, visual acuity, and intraocular pressure, taken two by two. The three clinical characteristics are not correlated (*P* > 0.05), neither in CG nor in DMCG. Additionally, for DMCG, we investigated the fasting glycemia (Table 1) and the retinal status (Figure 2B). Most patients (77.78%) present no retinopathy, however, as cataract formation hinders the visualization of the retina, the fundus is not visible in a few patients. Fasting glycemia is not correlated with visual acuity and intraocular pressure, but is positively correlated with age.

### miRNA quantification

We used qRT-PCR to evaluate the plasma levels of hsa-miR-19b, has-miR-34a, and hsa-miR-146a in DMCG patients (case group, *n* ═ 27) vs patients with cataract (control group, *n* ═ 23). Normalized Ct values (to cel-miR-39) follow a normal distribution. None of the three miRNAs correlate to any of the clinical characteristics of the patients (age, visual acuity, and intraocular pressure) (Table 2). The relative quantification experiment shows that the expression of hsa-miR-19b decreases in DMCG patients, while the expression of hsa-miR-34a and hsa-miR-146a increases (Table 3). However, only hsa-miR-34a and hsa-miR-146a changes are statistically significant.

We then investigated to what extent the expression profile of the three miRNAs varies depending on: (1) the aspect of the retina and (2) the site of opacification (Table 3). Firstly, concerning the retinal status, the findings presented for the entire case cohort hold true when examining the subgroup without retinopathy. This was expected, given that individuals with unaffected retina form the most well-represented sub-group (Figure 2B) in our case cohort (>75% of the entire group). As such, we observed significant upregulation for hsa-miR-34a and hsa-miR-146a, and insignificant downregulation for hsa-miR-19b in the subgroup with no retinopathy. However, while the direction of expression remains the same for the other two subgroups (mild retinopathy and fundus not visible), the statistical power is lost, ostensibly due to the reduced number of individuals in each subgroup. It is interesting to note that, when comparing the subgroup with moderate retinopathy to the one without retinopathy, we observe an overall increase in the fold change of hsa-miR-34a (3.5655 vs 1.9140) and, to a lesser extent, hsa-miR-146a (2.2086 vs 2.0742). Still, the meaningfulness of this finding is questionable, given its lack of statistical power.

Secondly, when investigating the miRNA expression profile based on the type of cataract (Figure 2A), similar results are obtained. We observe that although the stratification impacts the *P* value, the direction of expression remains the same as in the analysis of the complete cohort.

Lastly, we asked whether the dysregulation of the three miRNAs correlates with any of the clinical characteristics. As indicated by the Spearman coefficients in the correlation matrix, the expression levels of hsa-miR-19b, hsa-miR-34a, and hsa-miR-146a do not significantly associate with age, visual acuity, intraocular pressure, or fasting glycemia.

Our findings suggest the occurrence of DM in cataract alters the circulating level of hsa-miR-34a and hsa-miR-146a. However, we were unable to detect a differential expression profile based on the structural characteristics of the eye. Thus, despite revealing a similar pattern of miRNA expression within the subgroups, stratification reduces the analysis’s statistical power, presumably due to the effect of sample size.

## Discussion

In the present study, we made use of the sensitivity and specificity of the qRT-PCR technique to investigate the plasma expression levels of three miRNAs in patients with DM and cataract, compared to patients with cataract. We show that miR-34a and miR-146 are significantly upregulated in the case group, while the miR-19b changes are statistically irrelevant.

Our data on miR-34a confirm previous findings in age-related cataract [39, 44], conducted, notably, on lens or lens epithelial cells (LECs). In a case-control study on 110 patients, Chien et al. [39] described a positive correlation between the severity of cataract opacity and the level of miR-34a in LECs. Wu et al. [28] showed consistent upregulation of miR-34a in age-related cataractous human lenses. Lens development and cataractogenesis are impacted by apoptosis [45, 46], upregulation of miR-34 induces apoptosis in lenses and LECs by reducing the expression of anti-apoptotic proteins. In rat lenses, miR-34 triggers apoptosis and aggravate cataract by decreasing SIRT1 (thus elevating the expression of *P53*) [47]. In LECs under H_2_O_2_ exposure, miR-34a acts as a negative regulator of oxidative damage and apoptosis, as it lowers the levels of SIRT1, Bcl-2 [48], and GPX3 [49]. Under the same conditions, Wu et al. [50] showed that high expression of miR-34a-5p impairs the protective effects of circMED12L on LECs and correlates with a decrease in ALCAM. Age-induced expression of miR-34a in human cataractous LECs also stimulates mitochondria-mediated apoptosis by inhibiting Notch2 and activating caspase-9 [40]. On the other hand, overexpression of miR-34a was shown to inhibit the proliferation, migration, and epithelial–mesenchymal transition (EMT) of LECs, processes crucial for the development of posterior capsule opacification. In this regard, the main target molecules are c-Met (Akt and ERK1/2 pathway) [51], Notch1 (TGFβ2/Notch1 pathway) [52], and Snail1 (TGFβ/NEAT1/Snail1 pathway) [53].

In humans, although dysregulation of miR-34a (in plasma and blood mononuclear cells) was associated with DM [54, 55], few groups have studied the role of miR-34a in diabetic cataract. Interestingly, based on their analysis of posterior capsular tissue samples from patients with diabetic cataract and of LEC exposed to high glucose, Wang et al. [56] found that miR-34a acts as a mediator on the XIST - SMAD2 axis. Of note, the tissue expression data are in stark contrast with our results showing upregulation of miR-34a. Although lacking sufficient statistical power, our findings also imply the expression level of miR-34a is higher in patients with damaged retinal tissue. This raises questions regarding the tissue source of plasma miR-34a upregulation in DMCG patients.

While miR-146a has been shown to be differentially associated with DM pathology, there is an astounding lack of data regarding miR-146a’s involvement in cataract. In diabetic animal models, upregulation of miR-146a was noticed in mouse lens [42] and rat endothelial cells [57]. Interestingly, high glucose levels induce downregulation of miR-146a in the retinas of diabetic rats and in cultured endothelial cells from retinal microvessels and larger vessels, presumably through the regulatory effects of p300 [43]. In diabetic rat models, upregulated miR-146a inhibits NF-κB signaling pathway by targeting CARD10, IRAK1, and TRAF6, thus regulating the expression of important pro-inflammatory factors, such as TNF-α, IL-1β, and IL-6 [58, 59]. Moreover, given its association with cellular senescence, miR-146a has been proposed as an inflammaging miRNA and has been linked to mitochondrial (dys)function [60, 61]. By impacting NF-κB pathway, miR-146a also modulates antiviral response in the recipient cells and makes them more susceptible to infection [62].

Our study showed a rather expected (given the critical role inflammation plays in the pathogenesis of diabetic complications) increased expression of plasma miR-146a in DMCG patients [63–65]. In contrast, others found that circulant miR-146a downregulation is associated with DM type 2 susceptibility [66]. However, there is a lack of consistency regarding the direction of miR-146a changes in various tissues from diabetic patients. In diabetic cornea, miR-146a upregulation seems to be associated with delayed wound healing [41, 67], while in AH of diabetic patients, increased levels of miR-146a are associated with proliferative DR, although not statistically significant [35].

Except for one reference describing its presence in AH of patients with cataract, there are no data regarding miR-19b’s role in cataractogenesis [32]. However, miR-19b is dysregulated in several DM complications (diabetic nephropathy, cardiomyopathy, neuropathy, and retinopathy), with inflammation and apoptosis being the suspected underlying mechanisms [68–71]. In human retinal microvascular endothelial cells exposed to high glucose, lncRNA MEG3 inhibited the glucose-induced inflammation and apoptosis by regulating (through the JAK2/STAT3 signaling) the expression of miR-19b and its target, SOCS6 [72]. Another lncRNA, H19 also inhibits high glucose-induced inflammatory response by downregulating miR-19b and, thus, increasing SIRT1 expression in human retinal epithelial cells [70].

Together with other members of miR-17–1∼92a and miR-17–2∼363 clusters, miR-19b was shown to impact the pathogenesis of DR by modulating angiogenesis in diabetic rats [57]. miR-19b was found upregulated in the serum of DM type 1 patients (complicated with DR) [38] but downregulated in the AH of type 2 DM (complicated with non-proliferative DR and diabetic macular edema) [37]. In our lot of patients, plasma miR-19b is decreased, however, the data lacks statistical significance.

Our study has several limitations. We did not take into consideration certain factors that might have interfered with the plasma miRNA quantification, including the influence of different treatments of DM, comorbidities, body mass index, and lifestyle habits. Our study cohorts are relatively small, and this may have prevented us from observing associations that would be significant in larger cohorts. Finally, our findings are restricted to subjects of Caucasian origin, from Timis County of Romania.

## Conclusion

Our results show a significant upregulation of circulating miR-34a and miR-146 in patients with DM and cataract, compared with non-diabetic cataract controls. This suggests that two miRNAs are involved in distinct pathophysiological mechanisms of cataract formation, depending on the presence or the absence of DM. Scientific data show that miR-34a and miR-146a are expressed in various ocular tissues and extravascular fluids (LECs, retinal cells, cornea, and AH). Here, they are involved in apoptosis and inflammation, with various target molecules at stake (SIRT1, Notch1/2, IRAK1, and TRAF6). However, larger future studies are needed to validate our preliminary results and to determine the exact tissue origin of the two circulating miRNAs. This could help distinguish between the effects of miR-34a and miR-146 on the development of cataract in patients with DM, as opposed to those without DM.

## References

[1] Peterson SR, Silva PA, Murtha TJ, Sun JK (2018). Cataract surgery in patients with diabetes: management strategies. Semin Ophthalmol.

[2] Kiziltoprak H, Tekin K, Inanc M, Goker YS (2019). Cataract in diabetes mellitus. World J Diabetes.

[3] Schmid PWN, Lim NCH, Peters C (2021). Imbalances in the eye lens proteome are linked to cataract formation. Nat Struct Mol Biol.

[4] Satoh K, Takemura Y, Satoh M, Ozaki K, Kubota S (2021). Loss of FYCO1 leads to cataract formation. Sci Rep.

[5] Timsina R, Mainali L (2021). Association of alpha-crystallin with fiber cell plasma membrane of the eye lens accompanied by light scattering and cataract formation. Membranes (Basel).

[6] Ang MJ, Afshari NA (2021). Cataract and systemic disease: a review. Clin Exp Ophthalmol.

[7] Pratap T, Kokil P (2021). Efficient network selection for computer-aided cataract diagnosis under noisy environment. Comput Methods Programs Biomed.

[8] Pratap T, Kokil P (2019). Computer-aided diagnosis of cataract using deep transfer learning. Biomed Signal Process Control.

[9] Chen X, Xu J, Chen X, Yao K (2021). Cataract: advances in surgery and whether surgery remains the only treatment in future. Adv Ophthalmol Pract Res.

[10] Tognetto D, Brézin AP, Cummings AB, Malyugin BE, Kemer OE, Prieto I (2020). Rethinking elective cataract surgery diagnostics, assessments, and tools after the COVID-19 pandemic experience and beyond: insights from the EUROCOVCAT group. Diagnostics.

[11] Pershing S, Lum F, Hsu S, Kelly S, Chiang MF, Rich WL III (2020). Endophthalmitis after cataract surgery in the United States: a report from the intelligent research in sight registry, 2013–2017. Ophthalmology.

[12] Becker C, Schneider C, Aballéa S, Bailey C, Bourne R, Jick S (2018). Cataract in patients with diabetes mellitus—incidence rates in the U.K. and risk factors. Eye.

[13] Li L, Wan XH, Zhao GH (2014). Meta-analysis of the risk of cataract in type 2 diabetes. BMC Ophthalmol.

[14] Obrosova IG, Chung SSM, Kador PF (2010). Diabetic cataracts: mechanisms and management. Diabetes Metab Res Rev.

[15] Sayin N, Kara N, Pekel G (2015). Ocular complications of diabetes mellitus. World J Diabetes.

[16] Jeng CJ, Hsieh YT, Yang CM, Yang CH, Lin CL, Wang IJ (2018). Development of diabetic retinopathy after cataract surgery. PLoS One.

[17] Kim S Il, Kim SJ (2006). Prevalence and risk factors for cataracts in persons with type 2 diabetes mellitus. Korean J Ophthalmol.

[18] Dexheimer PJ, Cochella L (2020 Jun). MicroRNAs: from mechanism to organism. Front Cell Dev Biol.

[19] Saliminejad K, Khorram Khorshid HR, Soleymani Fard S, Ghaffari SH (2019). An overview of microRNAs: biology, functions, therapeutics, and analysis methods. J Cell Physiol.

[20] O’Brien J, Hayder H, Zayed Y, Peng C (2018 Aug). Overview of microRNA biogenesis, mechanisms of actions, and circulation. Front Endocrinol (Lausanne).

[21] Lin S, Gregory RI (2015). MicroRNA biogenesis pathways in cancer. Nat Rev Cancer.

[22] Wang E (2016). An overview of microRNA. RNA Technol Cardiovasc Med Res.

[23] Cui C, Cui Q (2020). The relationship of human tissue microRNAs with those from body fluids. Sci Rep.

[24] Montano M (2011). MicroRNAs: miRRORS of health and disease. Transl Res.

[25] Guay C, Roggli E, Nesca V, Jacovetti C, Regazzi R (2011). Diabetes mellitus, a microRNA-related disease?. Transl Res.

[26] De Candia P, Spinetti G, Specchia C, Sangalli E, La Sala L, Uccellatore A (2017). A unique plasma microRNA profile defines type 2 diabetes progression. PLoS One.

[27] Santovito D, Toto L, De Nardis V, Marcantonio P, D’Aloisio R, Mastropasqua A (2021). Plasma microRNA signature associated with retinopathy in patients with type 2 diabetes. Sci Rep.

[28] Wu C, Lin H, Wang Q, Chen W, Luo H, Chen W (2012). Discrepant expression of microRNAs in transparent and cataractous human lenses. Investig Ophthalmol Vis Sci.

[29] Tanaka Y, Tsuda S, Kunikata H, Sato J, Kokubun T, Yasuda M (2014). Profiles of extracellular miRNAs in the aqueous humor of glaucoma patients assessed with a microarray system. Sci Rep.

[30] Abdullah OA, El Gazzar WB, Salem TI, Elmohamady MN, Nasif SN, Eltaher SM (2019). miR-15a: a potential diagnostic biomarker and a candidate for non-operative therapeutic modality for age-related cataract. Br J Biomed Sci.

[31] Wu C, Liu Z, Ma L (2017). MiRNAs regulate oxidative stress related genes via binding to the 3’ UTR and TATA-box regions: a new hypothesis for cataract pathogenesis. BMC Ophthalmol.

[32] Dunmire JJ, Lagorous E, Bouhenni RA, Jones M, Edward DP (2013). MicroRNA in aqueous humor from patients with cataract. Exp Eye Res.

[33] Li Y, Liu S, Zhang F, Jiang P, Wu X, Liang Y (2015). Expression of the microRNAs hsa-miR-15a and hsa-miR-16-1 in lens epithelial cells of patients with age-related cataract. Int J Clin Exp Med.

[34] Yu X, Zheng H, Chan MTV, Wu WKK (2017). MicroRNAs: new players in cataract. Am J Transl Res.

[35] Chen S, Yuan M, Liu Y (2019). Landscape of microRNA in the aqueous humour of proliferative diabetic retinopathy as assessed by next-generation sequencing. Clin Exp Ophthalmol.

[36] Gao C, Fan F, Liu X, Yang J, Zhou X, Mei H (2021). Exosomal miRNA analysis of aqueous humour of diabetes and cataract patients. Curr Eye Res.

[37] Grieco GE, Sebastiani G, Eandi CM, Neri G, Nigi L, Brusco N (2020). MicroRNA expression in the aqueous humor of patients with diabetic macular edema. Int J Mol Sci.

[38] Liu HN, Cao NJ, Li X, Qian W, Chen XL (2018). Serum microRNA-211 as a biomarker for diabetic retinopathy via modulating Sirtuin 1. Biochem Biophys Res Commun.

[39] Chien K-H, Chen S-J, Liu J-H, Chang H-M, Woung L-C, Liang C-M (2013). Correlation between microRNA-34a levels and lens opacity severity in age-related cataracts. Eye.

[40] Fan F, Zhuang J, Zhou P, Liu X, Luo Y (2017). MicroRNA-34a promotes mitochondrial dysfunction-induced apoptosis in human lens epithelial cells by targeting Notch2. Oncotarget.

[41] Winkler MA, Dib C, Ljubimov AV, Saghizadeh M (2014). Targeting miR-146a to treat delayed wound healing in human diabetic organ-cultured corneas. PLoS One.

[42] Varma SD, Chandrasekaran K (2017). Micro RNA upregulation in diabetic lens. Invest Ophthalmol Vis Sci.

[43] Feng B, Chen S, McArthur K, Wu Y, Sen S, Ding Q (2011). miR-146a-mediated extracellular matrix protein production in chronic diabetes complications. Diabetes.

[44] Wei YL, Sun H (2019). Identification of hsa-mir-34a, hsa-mir-124, and hsa-mir-204 as signatures for cataract. J Cell Physiol.

[45] Kim B, Kim SY, Chung SK (2012). Changes in apoptosis factors in lens epithelial cells of cataract patients with diabetes mellitus. J Cataract Refract Surg.

[46] Yan Q, Liu JP, Wan-Cheng Li D (2006). Apoptosis in lens development and pathology. Differentiation.

[47] Xiu C, Jiang J, Song R (2019). Expression of miR-34a in cataract rats and its related mechanism. Exp Ther Med.

[48] Li QL, Zhang HY, Qin YJ, Meng QL, Yao XL, Guo HK (2016). MicroRNA-34a promoting apoptosis of human lens epithelial cells through down-regulation of B-cell lymphoma-2 and silent information regulator. Int J Ophthalmol.

[49] Wang S, Yu M, Yan H, Liu J, Guo C (2022). MiR-34a-5p negatively regulates oxidative stress on lens epithelial cells by silencing GPX3-A novel target. Curr Eye Res.

[50] Wu B, Sun Y, Hou J (2022). CircMED12L protects against hydrogen peroxide-induced apoptotic and oxidative injury in human lens epithelial cells by miR-34a-5p/ALCAM axis. Curr Eye Res.

[51] Feng D, Zhu N, Yu C, Lou D (2019 Apr). MicroRNA-34a suppresses human lens epithelial cell proliferation and migration via downregulation of c-Met. Clin Chim Acta.

[52] Han R, Hao P, Wang L, Li J, Shui S, Wang Y (2019). MicroRNA-34a inhibits epithelial-mesenchymal transition of lens epithelial cells by targeting Notch1. Exp Eye Res.

[53] Dong N (2020). Long noncoding RNA NEAT1 regulates TGF- β2-induced epithelial-mesenchymal transition of lens epithelial cells through the miR-34a/Snail1 and miR-204/Zeb1 pathways. Biomed Res Int.

[54] Shen Y, Xu H, Pan X, Wu W, Wang H, Yan L (2017). miR-34a and miR-125b are upregulated in peripheral blood mononuclear cells from patients with type 2 diabetes mellitus. Exp Ther Med.

[55] García-Jacobo RE, Uresti-Rivera EE, Portales-Pérez DP, González-Amaro R, Lara-Ramírez EE, Enciso-Moreno JA (2019). Circulating miR-146a, miR-34a and miR-375 in type 2 diabetes patients, pre-diabetic and normal-glycaemic individuals in relation to β-cell function, insulin resistance and metabolic parameters. Clin Exp Pharmacol Physiol.

[56] Wang C, Zhao R, Zhang S (2022). lncRNA XIST knockdown suppresses cell proliferation and promotes apoptosis in diabetic cataracts through the miR-34a/SMAD2 axis. Mol Med Rep.

[57] Kovacs B, Lumayag S, Cowan C, Xu S (2011). microRNAs in early diabetic retinopathy in streptozotocin-induced diabetic rats. Investig Ophthalmol Vis Sci.

[58] Yu HY, Meng LF, Lu XH, Liu LH, Ci X, Zhuo Z (2020). Protective effect of miR-146 against kidney injury in diabetic nephropathy rats through mediating the NF-κB signaling pathway. Eur Rev Med Pharmacol Sci.

[59] Zhuang P, Muraleedharan CK, Xu S (2017). Intraocular delivery of miR-146 inhibits diabetes-induced retinal functional defects in diabetic rat model. Investig Ophthalmol Vis Sci.

[60] Rippo MR, Olivieri F, Monsurrò V, Prattichizzo F, Albertini MC, Procopio AD (2014). MitomiRs in human inflammaging: a hypothesis involving miR-181a, miR-34a and miR-146a. Exp Gerontol.

[61] Rusca N, Monticelli S (2011). MiR-146a in immunity and disease. Mol Biol Int.

[62] Nahand JS, Karimzadeh MR, Nezamnia M, Fatemipour M, Khatami A, Jamshidi S (2020). The role of miR-146a in viral infection. IUBMB Life.

[63] Bhatt K, Lanting LL, Jia Y, Yadav S, Reddy MA, Nathaniel M (2016). Anti-inflammatory role of microRNA-146a in the pathogenesis of diabetic nephropathy. J Am Soc Nephrol.

[64] Wang L, Chopp M, Szalad A, Zhang Y, Wang X, Zhang RL (2014). The role of miR-146a in dorsal root ganglia neurons of experimental diabetic peripheral neuropathy. Neuroscience.

[65] Balasubramanyam M, Aravind S, Gokulakrishnan K, Prabu P, Sathishkumar C, Ranjani H (2011). Impaired miR-146a expression links subclinical inflammation and insulin resistance in type 2 diabetes. Mol Cell Biochem.

[66] Alipoor B, Ghaedi H, Meshkani R, Torkamandi S, Sana Saffari S, Iranpour M (2017). Association of MiR-146a expression and type 2 diabetes mellitus: a meta-analysis. Int J Mol Cell Med.

[67] Poe AJ, Shah R, Khare D, Kulkarni M, Phan H, Ghiam S (2022). Regulatory role of miR-146a in corneal epithelial wound healing via its inflammatory targets in human diabetic cornea. Ocul Surf.

[68] Copier CU, León L, Fernández M, Contador D, Calligaris SD (2017). Circulating miR-19b and miR-181b are potential biomarkers for diabetic cardiomyopathy. Sci Rep.

[69] Rajabinejad M, Asadi G, Ranjbar S, Varmaziar FR, Karimi M, Salari F (2022 Mar). The MALAT1-H19/miR-19b-3p axis can be a fingerprint for diabetic neuropathy. Immunol Lett.

[70] Luo R, Li L, Hu YX, Xiao F (2021). LncRNA H19 inhibits high glucose-induced inflammatory responses of human retinal epithelial cells by targeting miR-19b to increase SIRT1 expression. Kaohsiung J Med Sci.

[71] Lv LL, Feng Y, Wu M, Wang B, Li Z-L, Zhong X (2020). Exosomal miRNA-19b-3p of tubular epithelial cells promotes M1 macrophage activation in kidney injury. Cell Death Differ.

[72] Xiao F, Li L, Fu JS, Hu YX, Luo R (2020). Regulation of the miR-19b-mediated SOCS6-JAK2/STAT3 pathway by lncRNA MEG3 is involved in high glucose-induced apoptosis in hRMECs. Biosci Rep.

